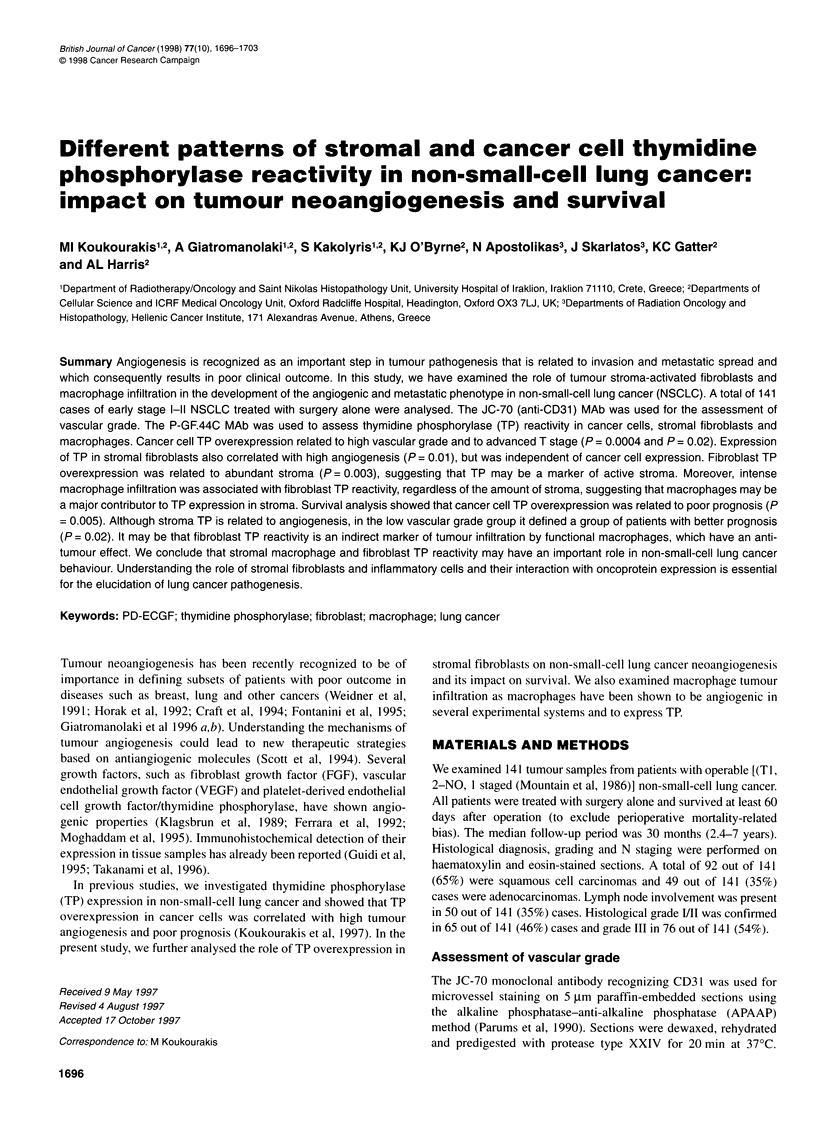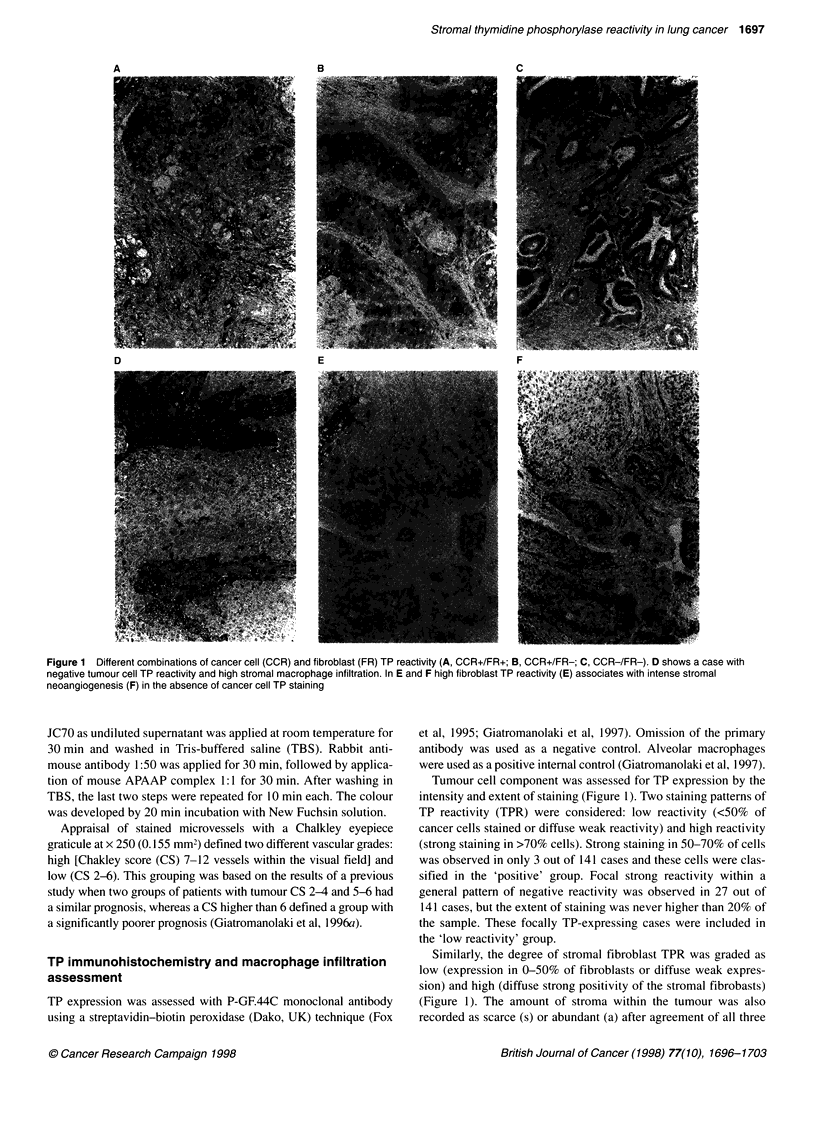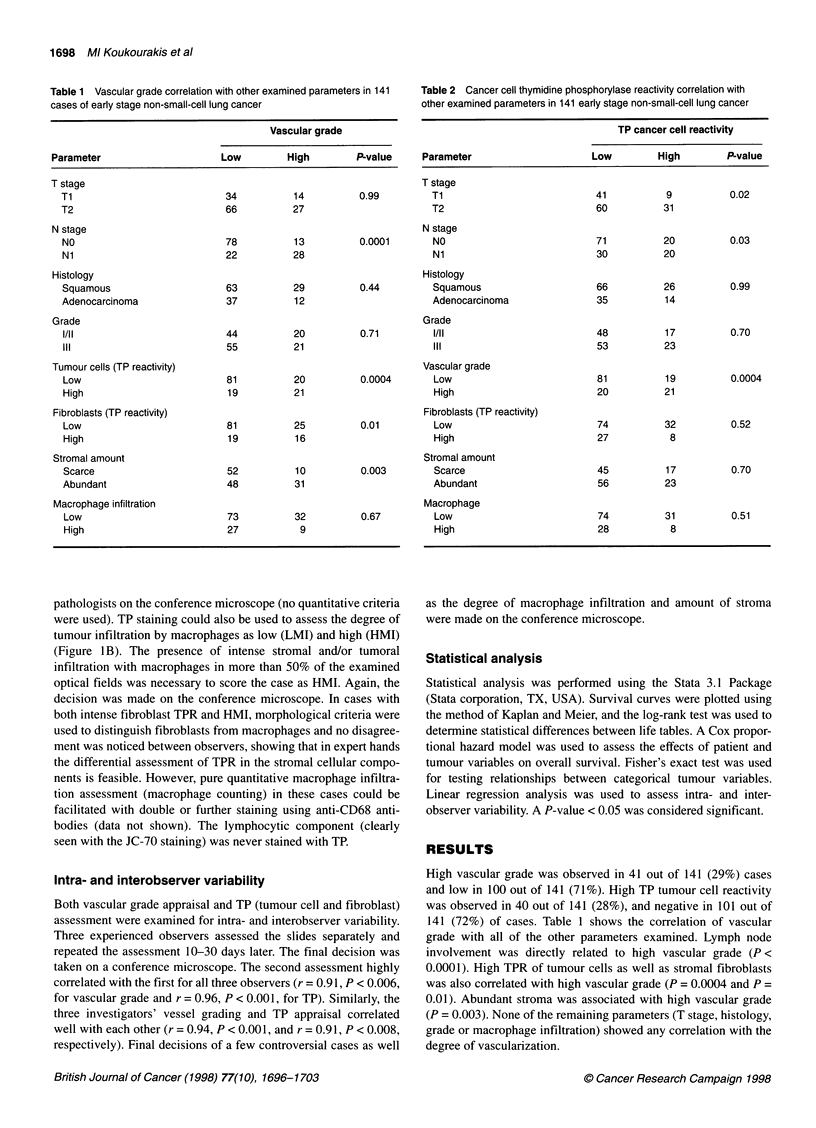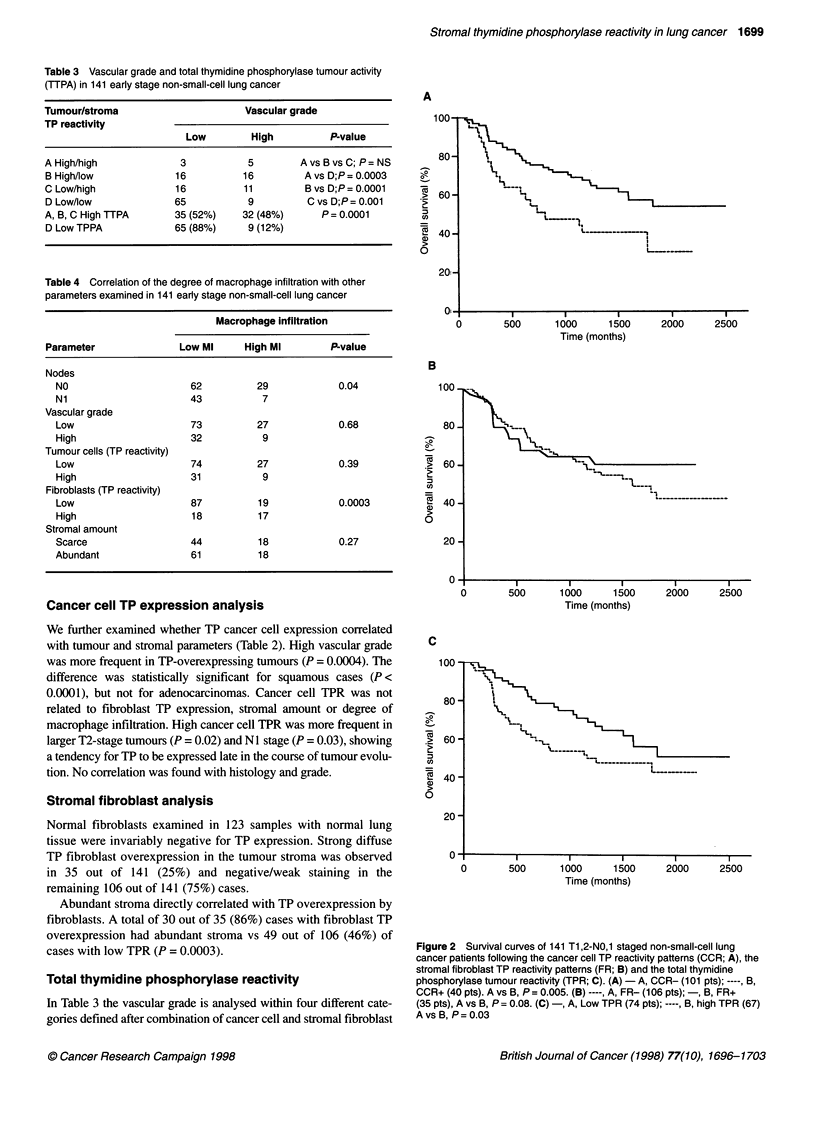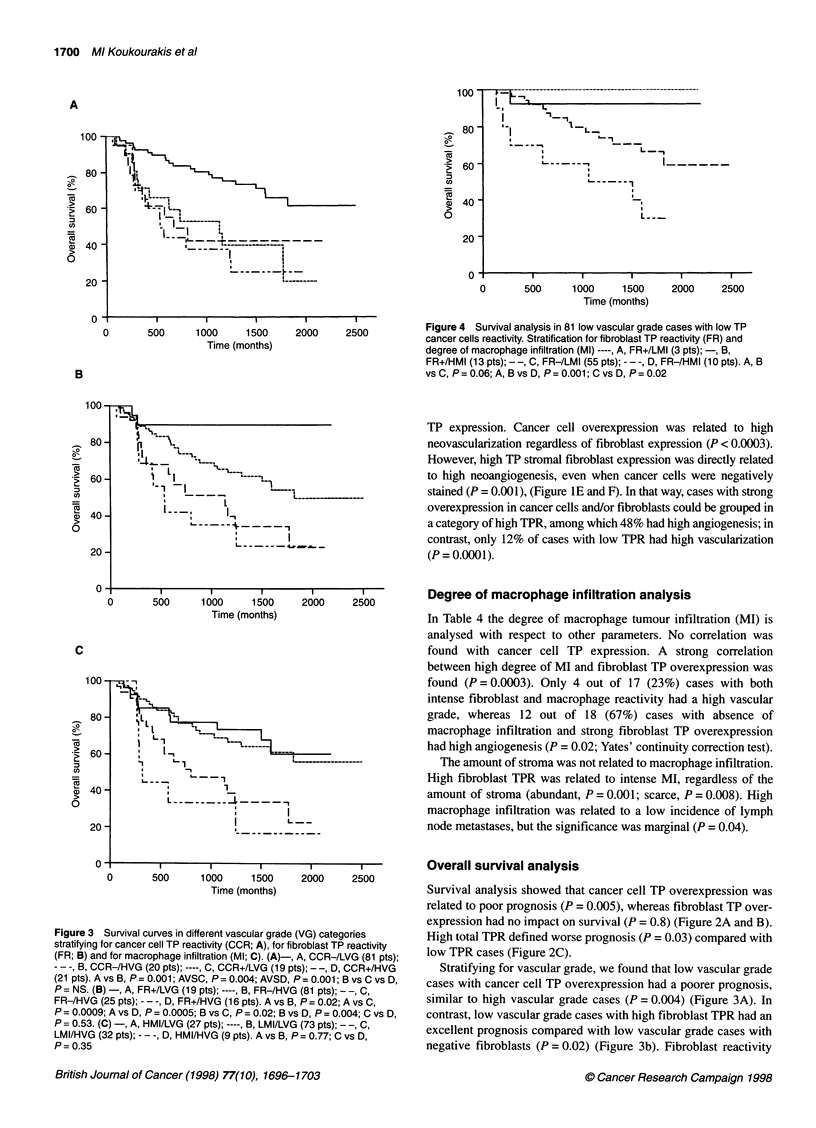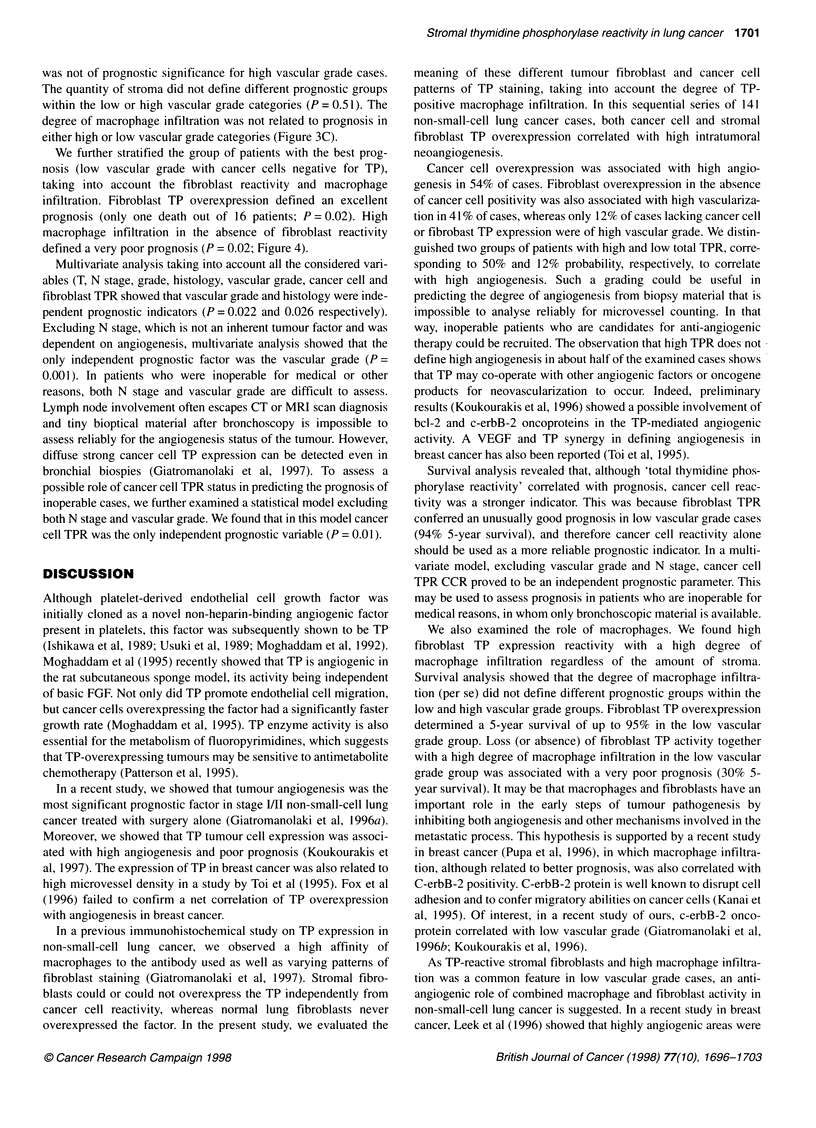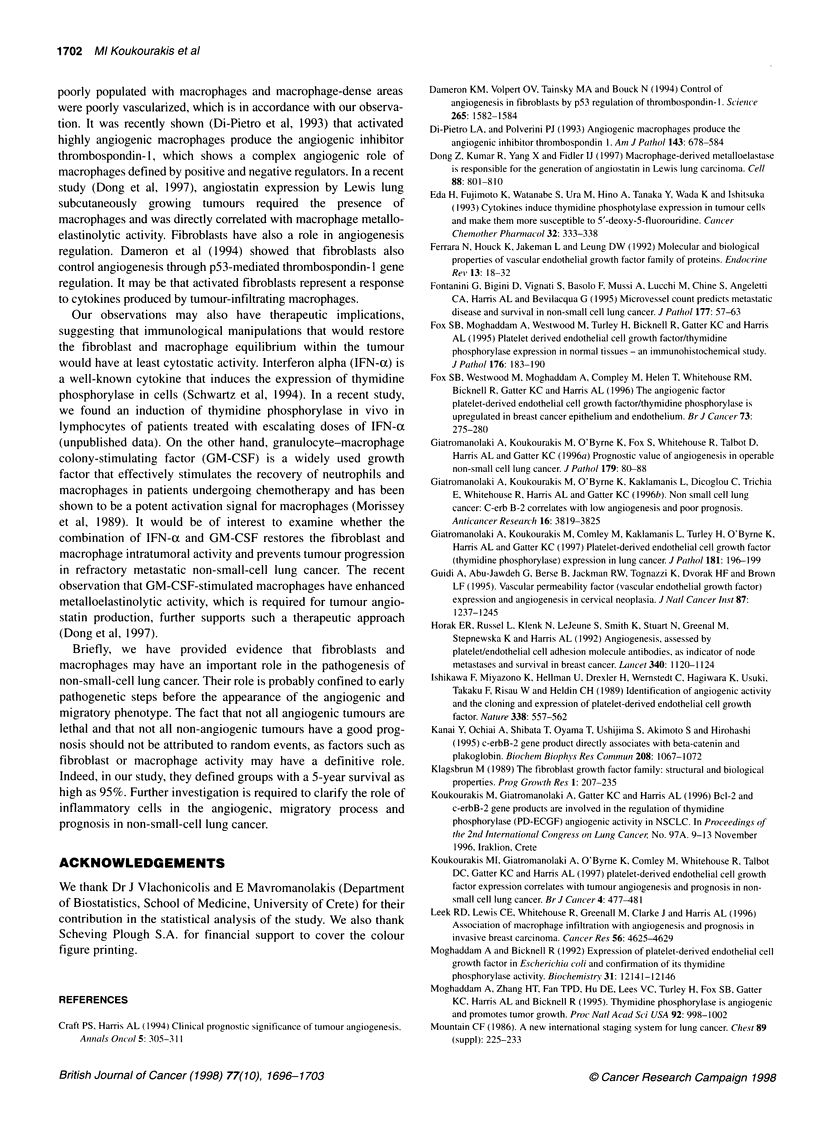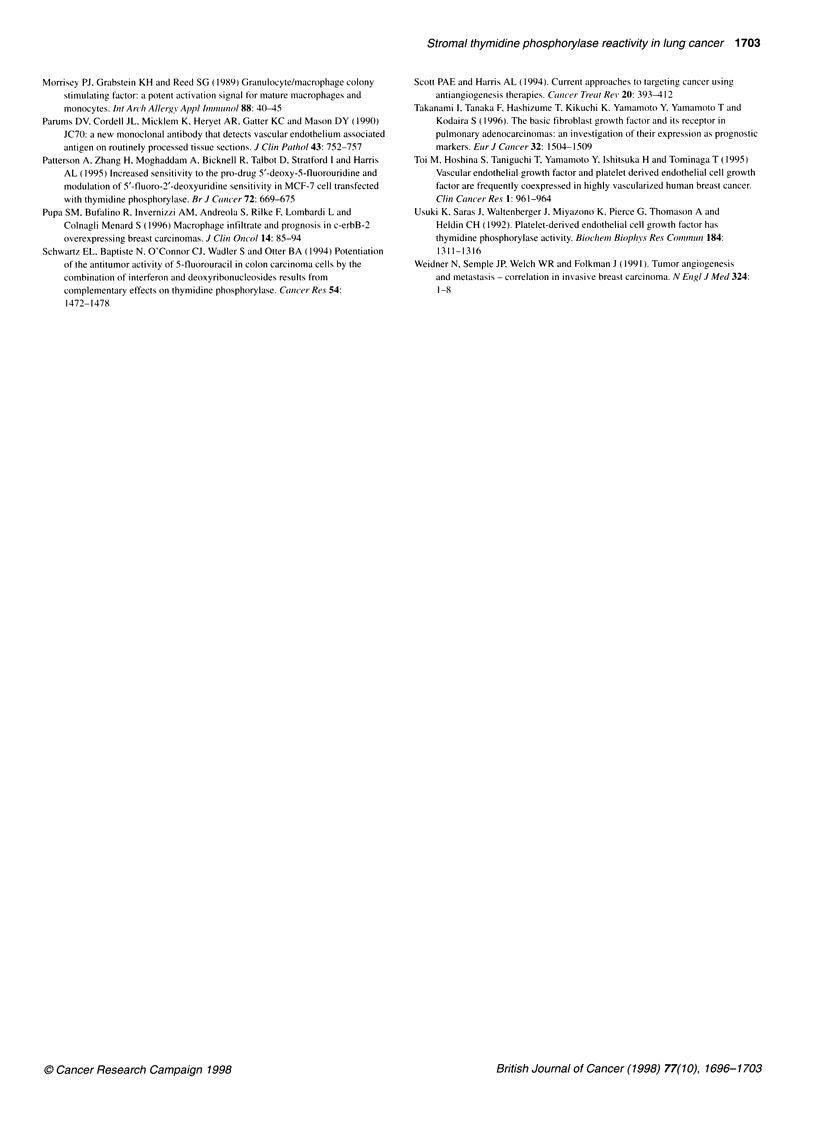# Different patterns of stromal and cancer cell thymidine phosphorylase reactivity in non-small-cell lung cancer: impact on tumour neoangiogenesis and survival.

**DOI:** 10.1038/bjc.1998.280

**Published:** 1998-05

**Authors:** M. I. Koukourakis, A. Giatromanolaki, S. Kakolyris, K. J. O'Byrne, N. Apostolikas, J. Skarlatos, K. C. Gatter, A. L. Harris

**Affiliations:** Department of Radiotherapy/Oncology and Saint Nikolas Histopathology Unit, University Hospital of Iraklion, Crete, Greece.

## Abstract

**Images:**


British Journal of Cancer (1998) 77(10), 1696–1703
© 1998 Cancer Research Campaign

### Different patterns of stromal and cancer cell thymidine phosphorylase reactivity in non-small-cell lung cancer: impact on tumour neoangiogenesis and survival

**MI Koukourakis[1,2], A Giatromanolaki[1,2], S Kakolyris[1,2], KJ O'Byrne[2], N Apostolikas[3], J Skarlatos[3], KC Gatter[2] and AL Harris[2]**

[1]Department of Radiotherapy/Oncology and Saint Nikolas Histopathology Unit, University Hospital of Iraklion, Iraklion 71110, Crete, Greece; [2]Departments of Cellular Science and ICRF Medical Oncology Unit, Oxford Radcliffe Hospital, Headington, Oxford OX3 7LJ, UK; [3]Departments of Radiation Oncology and Histopathology, Hellenic Cancer Institute, 171 Alexandras Avenue, Athens, Greece

**Summary** Angiogenesis is recognized as an important step in tumour pathogenesis that is related to invasion and metastatic spread and which consequently results in poor clinical outcome. In this study, we have examined the role of tumour stroma-activated fibroblasts and macrophage infiltration in the development of the angiogenic and metastatic phenotype in non-small-cell lung cancer (NSCLC). A total of 141 cases of early stage I–II NSCLC treated with surgery alone were analysed. The JC-70 (anti-CD31) MAb was used for the assessment of vascular grade. The P-GF.44C MAb was used to assess thymidine phosphorylase (TP) reactivity in cancer cells, stromal fibroblasts and macrophages. Cancer cell TP overexpression related to high vascular grade and to advanced T stage ($P = 0.0004$ and $P = 0.02$). Expression of TP in stromal fibroblasts also correlated with high angiogenesis ($P = 0.01$), but was independent of cancer cell expression. Fibroblast TP overexpression was related to abundant stroma ($P = 0.003$), suggesting that TP may be a marker of active stroma. Moreover, intense macrophage infiltration was associated with fibroblast TP reactivity, regardless of the amount of stroma, suggesting that macrophages may be a major contributor to TP expression in stroma. Survival analysis showed that cancer cell TP overexpression was related to poor prognosis ($P = 0.005$). Although stroma TP is related to angiogenesis, in the low vascular grade group it defined a group of patients with better prognosis ($P = 0.02$). It may be that fibroblast TP reactivity is an indirect marker of tumour infiltration by functional macrophages, which have an anti-tumour effect. We conclude that stromal macrophage and fibroblast TP reactivity may have an important role in non-small-cell lung cancer behaviour. Understanding the role of stromal fibroblasts and inflammatory cells and their interaction with oncoprotein expression is essential for the elucidation of lung cancer pathogenesis.

**Keywords:** PD-ECGF; thymidine phosphorylase; fibroblast; macrophage; lung cancer

Tumour neoangiogenesis has been recently recognized to be of importance in defining subsets of patients with poor outcome in diseases such as breast, lung and other cancers (Weidner et al, 1991; Horak et al, 1992; Craft et al, 1994; Fontanini et al, 1995; Giatromanolaki et al 1996 *a,b*). Understanding the mechanisms of tumour angiogenesis could lead to new therapeutic strategies based on antiangiogenic molecules (Scott et al, 1994). Several growth factors, such as fibroblast growth factor (FGF), vascular endothelial growth factor (VEGF) and platelet-derived endothelial cell growth factor/thymidine phosphorylase, have shown angiogenic properties (Klagsbrun et al, 1989; Ferrara et al, 1992; Moghaddam et al, 1995). Immunohistochemical detection of their expression in tissue samples has already been reported (Guidi et al, 1995; Takanami et al, 1996).

In previous studies, we investigated thymidine phosphorylase (TP) expression in non-small-cell lung cancer and showed that TP overexpression in cancer cells was correlated with high tumour angiogenesis and poor prognosis (Koukourakis et al, 1997). In the present study, we further analysed the role of TP overexpression in stromal fibroblasts on non-small-cell lung cancer neoangiogenesis and its impact on survival. We also examined macrophage tumour infiltration as macrophages have been shown to be angiogenic in several experimental systems and to express TP.

*Received 9 May 1997*
*Revised 4 August 1997*
*Accepted 17 October 1997*

*Correspondence to:* M Koukourakis

### MATERIALS AND METHODS

We examined 141 tumour samples from patients with operable [(T1, 2–NO, 1 staged (Mountain et al, 1986)] non-small-cell lung cancer. All patients were treated with surgery alone and survived at least 60 days after operation (to exclude perioperative mortality-related bias). The median follow-up period was 30 months (2.4–7 years). Histological diagnosis, grading and N staging were performed on haematoxylin and eosin-stained sections. A total of 92 out of 141 (65%) were squamous cell carcinomas and 49 out of 141 (35%) cases were adenocarcinomas. Lymph node involvement was present in 50 out of 141 (35%) cases. Histological grade I/II was confirmed in 65 out of 141 (46%) cases and grade III in 76 out of 141 (54%).

### Assessment of vascular grade

The JC-70 monoclonal antibody recognizing CD31 was used for microvessel staining on 5 μm paraffin-embedded sections using the alkaline phosphatase–anti-alkaline phosphatase (APAAP) method (Parums et al, 1990). Sections were dewaxed, rehydrated and predigested with protease type XXIV for 20 min at 37°C.

**Figure 1** Different combinations of cancer cell (CCR) and fibroblast (FR) TP reactivity (**A**, CCR+/FR+; **B**, CCR+/FR–; **C**, CCR–/FR–). **D** shows a case with negative tumour cell TP reactivity and high stromal macrophage infiltration. In **E** and **F** high fibroblast TP reactivity (**E**) associates with intense stromal neoangiogenesis (**F**) in the absence of cancer cell TP staining

JC70 as undiluted supernatant was applied at room temperature for 30 min and washed in Tris-buffered saline (TBS). Rabbit anti-mouse antibody 1:50 was applied for 30 min, followed by application of mouse APAAP complex 1:1 for 30 min. After washing in TBS, the last two steps were repeated for 10 min each. The colour was developed by 20 min incubation with New Fuchsin solution.

Appraisal of stained microvessels with a Chalkley eyepiece graticule at × 250 (0.155 $mm^2$) defined two different vascular grades: high [Chakley score (CS) 7–12 vessels within the visual field] and low (CS 2–6). This grouping was based on the results of a previous study when two groups of patients with tumour CS 2–4 and 5–6 had a similar prognosis, whereas a CS higher than 6 defined a group with a significantly poorer prognosis (Giatromanolaki et al, 1996*a*).

### TP immunohistochemistry and macrophage infiltration assessment

TP expression was assessed with P-GF.44C monoclonal antibody using a streptavidin–biotin peroxidase (Dako, UK) technique (Fox et al, 1995; Giatromanolaki et al, 1997). Omission of the primary antibody was used as a negative control. Alveolar macrophages were used as a positive internal control (Giatromanolaki et al, 1997).

Tumour cell component was assessed for TP expression by the intensity and extent of staining (Figure 1). Two staining patterns of TP reactivity (TPR) were considered: low reactivity (<50% of cancer cells stained or diffuse weak reactivity) and high reactivity (strong staining in >70% cells). Strong staining in 50–70% of cells was observed in only 3 out of 141 cases and these cells were classified in the ‘positive’ group. Focal strong reactivity within a general pattern of negative reactivity was observed in 27 out of 141 cases, but the extent of staining was never higher than 20% of the sample. These focally TP-expressing cases were included in the ‘low reactivity’ group.

Similarly, the degree of stromal fibroblast TPR was graded as low (expression in 0–50% of fibroblasts or diffuse weak expression) and high (diffuse strong positivity of the stromal fibrobasts) (Figure 1). The amount of stroma within the tumour was also recorded as scarce (s) or abundant (a) after agreement of all three

© Cancer Research Campaign 1998

**Table 1** Vascular grade correlation with other examined parameters in 141 cases of early stage non-small-cell lung cancer

| Parameter | Vascular grade | | |
|---|---|---|---|
| | Low | High | P-value |
| T stage | | | |
| T1 | 34 | 14 | 0.99 |
| T2 | 66 | 27 | |
| N stage | | | |
| N0 | 78 | 13 | 0.0001 |
| N1 | 22 | 28 | |
| Histology | | | |
| Squamous | 63 | 29 | 0.44 |
| Adenocarcinoma | 37 | 12 | |
| Grade | | | |
| I/II | 44 | 20 | 0.71 |
| III | 55 | 21 | |
| Tumour cells (TP reactivity) | | | |
| Low | 81 | 20 | 0.0004 |
| High | 19 | 21 | |
| Fibroblasts (TP reactivity) | | | |
| Low | 81 | 25 | 0.01 |
| High | 19 | 16 | |
| Stromal amount | | | |
| Scarce | 52 | 10 | 0.003 |
| Abundant | 48 | 31 | |
| Macrophage infiltration | | | |
| Low | 73 | 32 | 0.67 |
| High | 27 | 9 | |

**Table 2** Cancer cell thymidine phosphorylase reactivity correlation with other examined parameters in 141 early stage non-small-cell lung cancer

| Parameter | TP cancer cell reactivity | | |
|---|---|---|---|
| | Low | High | P-value |
| T stage | | | |
| T1 | 41 | 9 | 0.02 |
| T2 | 60 | 31 | |
| N stage | | | |
| N0 | 71 | 20 | 0.03 |
| N1 | 30 | 20 | |
| Histology | | | |
| Squamous | 66 | 26 | 0.99 |
| Adenocarcinoma | 35 | 14 | |
| Grade | | | |
| I/II | 48 | 17 | 0.70 |
| III | 53 | 23 | |
| Vascular grade | | | |
| Low | 81 | 19 | 0.0004 |
| High | 20 | 21 | |
| Fibroblasts (TP reactivity) | | | |
| Low | 74 | 32 | 0.52 |
| High | 27 | 8 | |
| Stromal amount | | | |
| Scarce | 45 | 17 | 0.70 |
| Abundant | 56 | 23 | |
| Macrophage | | | |
| Low | 74 | 31 | 0.51 |
| High | 28 | 8 | |

pathologists on the conference microscope (no quantitative criteria were used). TP staining could also be used to assess the degree of tumour infiltration by macrophages as low (LMI) and high (HMI) (Figure 1B). The presence of intense stromal and/or tumoral infiltration with macrophages in more than 50% of the examined optical fields was necessary to score the case as HMI. Again, the decision was made on the conference microscope. In cases with both intense fibroblast TPR and HMI, morphological criteria were used to distinguish fibroblasts from macrophages and no disagreement was noticed between observers, showing that in expert hands the differential assessment of TPR in the stromal cellular components is feasible. However, pure quantitative macrophage infiltration assessment (macrophage counting) in these cases could be facilitated with double or further staining using anti-CD68 antibodies (data not shown). The lymphocytic component (clearly seen with the JC-70 staining) was never stained with TP.

### Intra- and interobserver variability

Both vascular grade appraisal and TP (tumour cell and fibroblast) assessment were examined for intra- and interobserver variability. Three experienced observers assessed the slides separately and repeated the assessment 10–30 days later. The final decision was taken on a conference microscope. The second assessment highly correlated with the first for all three observers ($r = 0.91$, $P < 0.006$, for vascular grade and $r = 0.96$, $P < 0.001$, for TP). Similarly, the three investigators' vessel grading and TP appraisal correlated well with each other ($r = 0.94$, $P < 0.001$, and $r = 0.91$, $P < 0.008$, respectively). Final decisions of a few controversial cases as well as the degree of macrophage infiltration and amount of stroma were made on the conference microscope.

### Statistical analysis

Statistical analysis was performed using the Stata 3.1 Package (Stata corporation, TX, USA). Survival curves were plotted using the method of Kaplan and Meier, and the log-rank test was used to determine statistical differences between life tables. A Cox proportional hazard model was used to assess the effects of patient and tumour variables on overall survival. Fisher's exact test was used for testing relationships between categorical tumour variables. Linear regression analysis was used to assess intra- and inter-observer variability. A $P$-value < 0.05 was considered significant.

### RESULTS

High vascular grade was observed in 41 out of 141 (29%) cases and low in 100 out of 141 (71%). High TP tumour cell reactivity was observed in 40 out of 141 (28%), and negative in 101 out of 141 (72%) of cases. Table 1 shows the correlation of vascular grade with all of the other parameters examined. Lymph node involvement was directly related to high vascular grade ($P <$ 0.0001). High TPR of tumour cells as well as stromal fibroblasts was also correlated with high vascular grade ($P = 0.0004$ and $P =$ 0.01). Abundant stroma was associated with high vascular grade ($P = 0.003$). None of the remaining parameters (T stage, histology, grade or macrophage infiltration) showed any correlation with the degree of vascularization.

© Cancer Research Campaign 1998

**Table 3** Vascular grade and total thymidine phosphorylase tumour activity (TTPA) in 141 early stage non-small-cell lung cancer

| Tumour/stroma TP reactivity | Vascular grade | | |
|---|---|---|---|
| | Low | High | *P*-value |
| A High/high | 3 | 5 | A vs B vs C; $P$ = NS |
| B High/low | 16 | 16 | A vs D; $P$ = 0.0003 |
| C Low/high | 16 | 11 | B vs D; $P$ = 0.0001 |
| D Low/low | 65 | 9 | C vs D; $P$ = 0.001 |
| A, B, C High TTPA | 35 (52%) | 32 (48%) | $P$ = 0.0001 |
| D Low TPPA | 65 (88%) | 9 (12%) | |

**Table 4** Correlation of the degree of macrophage infiltration with other parameters examined in 141 early stage non-small-cell lung cancer

| Parameter | Macrophage infiltration | | |
|---|---|---|---|
| | Low MI | High MI | *P*-value |
| Nodes | | | |
| N0 | 62 | 29 | 0.04 |
| N1 | 43 | 7 | |
| Vascular grade | | | |
| Low | 73 | 27 | 0.68 |
| High | 32 | 9 | |
| Tumour cells (TP reactivity) | | | |
| Low | 74 | 27 | 0.39 |
| High | 31 | 9 | |
| Fibroblasts (TP reactivity) | | | |
| Low | 87 | 19 | 0.0003 |
| High | 18 | 17 | |
| Stromal amount | | | |
| Scarce | 44 | 18 | 0.27 |
| Abundant | 61 | 18 | |

### Cancer cell TP expression analysis

We further examined whether TP cancer cell expression correlated with tumour and stromal parameters (Table 2). High vascular grade was more frequent in TP-overexpressing tumours ($P$ = 0.0004). The difference was statistically significant for squamous cases ($P$ < 0.0001), but not for adenocarcinomas. Cancer cell TPR was not related to fibroblast TP expression, stromal amount or degree of macrophage infiltration. High cancer cell TPR was more frequent in larger T2-stage tumours ($P$ = 0.02) and N1 stage ($P$ = 0.03), showing a tendency for TP to be expressed late in the course of tumour evolution. No correlation was found with histology and grade.

### Stromal fibroblast analysis

Normal fibroblasts examined in 123 samples with normal lung tissue were invariably negative for TP expression. Strong diffuse TP fibroblast overexpression in the tumour stroma was observed in 35 out of 141 (25%) and negative/weak staining in the remaining 106 out of 141 (75%) cases.

Abundant stroma directly correlated with TP overexpression by fibroblasts. A total of 30 out of 35 (86%) cases with fibroblast TP overexpression had abundant stroma vs 49 out of 106 (46%) of cases with low TPR ($P$ = 0.0003).

### Total thymidine phosphorylase reactivity

In Table 3 the vascular grade is analysed within four different categories defined after combination of cancer cell and stromal fibroblast

**Figure 2** Survival curves of 141 T1,2-N0,1 staged non-small-cell lung cancer patients following the cancer cell TP reactivity patterns (CCR; **A**), the stromal fibroblast TP reactivity patterns (FR; **B**) and the total thymidine phosphorylase tumour reactivity (TPR; **C**). (**A**) — A, CCR– (101 pts); ----, B, CCR+ (40 pts). A vs B, $P$ = 0.005. (**B**) ----, A, FR– (106 pts); —, B, FR+ (35 pts), A vs B, $P$ = 0.08. (**C**) —, A, Low TPR (74 pts); ----, B, high TPR (67) A vs B, $P$ = 0.03

**Figure 3** Survival curves in different vascular grade (VG) categories stratifying for cancer cell TP reactivity (CCR; **A**), for fibroblast TP reactivity (FR; **B**) and for macrophage infiltration (MI; **C**). (**A**)—, A, CCR–/LVG (81 pts); - - -, B, CCR–/HVG (20 pts); ----, C, CCR+/LVG (19 pts); – –, D, CCR+/HVG (21 pts). A vs B, $P = 0.001$; AVSC, $P = 0.004$; AVSD, $P = 0.001$; B vs C vs D, $P =$ NS. (**B**) —, A, FR+/LVG (19 pts); ----, B, FR–/HVG (81 pts); – –, C, FR–/HVG (25 pts); - - -, D, FR+/HVG (16 pts). A vs B, $P = 0.02$; A vs C, $P = 0.0009$; A vs D, $P = 0.0005$; B vs C, $P = 0.02$; B vs D, $P = 0.004$; C vs D, $P = 0.53$. (**C**) —, A, HMI/LVG (27 pts); ----, B, LMI/LVG (73 pts); – –, C, LMI/HVG (32 pts); - - -, D, HMI/HVG (9 pts). A vs B, $P = 0.77$; C vs D, $P = 0.35$

**Figure 4** Survival analysis in 81 low vascular grade cases with low TP cancer cells reactivity. Stratification for fibroblast TP reactivity (FR) and degree of macrophage infiltration (MI) ----, A, FR+/LMI (3 pts); —, B, FR+/HMI (13 pts); – –, C, FR–/LMI (55 pts); - - -, D, FR–/HMI (10 pts). A, B vs C, $P = 0.06$; A, B vs D, $P = 0.001$; C vs D, $P = 0.02$

TP expression. Cancer cell overexpression was related to high neovascularization regardless of fibroblast expression ($P < 0.0003$). However, high TP stromal fibroblast expression was directly related to high neoangiogenesis, even when cancer cells were negatively stained ($P = 0.001$), (Figure 1E and F). In that way, cases with strong overexpression in cancer cells and/or fibroblasts could be grouped in a category of high TPR, among which 48% had high angiogenesis; in contrast, only 12% of cases with low TPR had high vascularization ($P = 0.0001$).

### Degree of macrophage infiltration analysis

In Table 4 the degree of macrophage tumour infiltration (MI) is analysed with respect to other parameters. No correlation was found with cancer cell TP expression. A strong correlation between high degree of MI and fibroblast TP overexpression was found ($P = 0.0003$). Only 4 out of 17 (23%) cases with both intense fibroblast and macrophage reactivity had a high vascular grade, whereas 12 out of 18 (67%) cases with absence of macrophage infiltration and strong fibroblast TP overexpression had high angiogenesis ($P = 0.02$; Yates' continuity correction test).

The amount of stroma was not related to macrophage infiltration. High fibroblast TPR was related to intense MI, regardless of the amount of stroma (abundant, $P = 0.001$; scarce, $P = 0.008$). High macrophage infiltration was related to a low incidence of lymph node metastases, but the significance was marginal ($P = 0.04$).

### Overall survival analysis

Survival analysis showed that cancer cell TP overexpression was related to poor prognosis ($P = 0.005$), whereas fibroblast TP overexpression had no impact on survival ($P = 0.8$) (Figure 2A and B). High total TPR defined worse prognosis ($P = 0.03$) compared with low TPR cases (Figure 2C).

Stratifying for vascular grade, we found that low vascular grade cases with cancer cell TP overexpression had a poorer prognosis, similar to high vascular grade cases ($P = 0.004$) (Figure 3A). In contrast, low vascular grade cases with high fibroblast TPR had an excellent prognosis compared with low vascular grade cases with negative fibroblasts ($P = 0.02$) (Figure 3b). Fibroblast reactivity

© Cancer Research Campaign 1998

was not of prognostic significance for high vascular grade cases. The quantity of stroma did not define different prognostic groups within the low or high vascular grade categories ($P = 0.51$). The degree of macrophage infiltration was not related to prognosis in either high or low vascular grade categories (Figure 3C).

We further stratified the group of patients with the best prognosis (low vascular grade with cancer cells negative for TP), taking into account the fibroblast reactivity and macrophage infiltration. Fibroblast TP overexpression defined an excellent prognosis (only one death out of 16 patients; $P = 0.02$). High macrophage infiltration in the absence of fibroblast reactivity defined a very poor prognosis ($P = 0.02$; Figure 4).

Multivariate analysis taking into account all the considered variables (T, N stage, grade, histology, vascular grade, cancer cell and fibroblast TPR showed that vascular grade and histology were independent prognostic indicators ($P = 0.022$ and 0.026 respectively). Excluding N stage, which is not an inherent tumour factor and was dependent on angiogenesis, multivariate analysis showed that the only independent prognostic factor was the vascular grade ($P = 0.001$). In patients who were inoperable for medical or other reasons, both N stage and vascular grade are difficult to assess. Lymph node involvement often escapes CT or MRI scan diagnosis and tiny bioptical material after bronchoscopy is impossible to assess reliably for the angiogenesis status of the tumour. However, diffuse strong cancer cell TP expression can be detected even in bronchial biospies (Giatromanolaki et al, 1997). To assess a possible role of cancer cell TPR status in predicting the prognosis of inoperable cases, we further examined a statistical model excluding both N stage and vascular grade. We found that in this model cancer cell TPR was the only independent prognostic variable ($P = 0.01$).

### DISCUSSION

Although platelet-derived endothelial cell growth factor was initially cloned as a novel non-heparin-binding angiogenic factor present in platelets, this factor was subsequently shown to be TP (Ishikawa et al, 1989; Usuki et al, 1989; Moghaddam et al, 1992). Moghaddam et al (1995) recently showed that TP is angiogenic in the rat subcutaneous sponge model, its activity being independent of basic FGF. Not only did TP promote endothelial cell migration, but cancer cells overexpressing the factor had a significantly faster growth rate (Moghaddam et al, 1995). TP enzyme activity is also essential for the metabolism of fluoropyrimidines, which suggests that TP-overexpressing tumours may be sensitive to antimetabolite chemotherapy (Patterson et al, 1995).

In a recent study, we showed that tumour angiogenesis was the most significant prognostic factor in stage I/II non-small-cell lung cancer treated with surgery alone (Giatromanolaki et al, 1996*a*). Moreover, we showed that TP tumour cell expression was associated with high angiogenesis and poor prognosis (Koukourakis et al, 1997). The expression of TP in breast cancer was also related to high microvessel density in a study by Toi et al (1995). Fox et al (1996) failed to confirm a net correlation of TP overexpression with angiogenesis in breast cancer.

In a previous immunohistochemical study on TP expression in non-small-cell lung cancer, we observed a high affinity of macrophages to the antibody used as well as varying patterns of fibroblast staining (Giatromanolaki et al, 1997). Stromal fibroblasts could or could not overexpress the TP independently from cancer cell reactivity, whereas normal lung fibroblasts never overexpressed the factor. In the present study, we evaluated the meaning of these different tumour fibroblast and cancer cell patterns of TP staining, taking into account the degree of TP-positive macrophage infiltration. In this sequential series of 141 non-small-cell lung cancer cases, both cancer cell and stromal fibroblast TP overexpression correlated with high intratumoral neoangiogenesis.

Cancer cell overexpression was associated with high angiogenesis in 54% of cases. Fibroblast overexpression in the absence of cancer cell positivity was also associated with high vascularization in 41% of cases, whereas only 12% of cases lacking cancer cell or fibrobast TP expression were of high vascular grade. We distinguished two groups of patients with high and low total TPR, corresponding to 50% and 12% probability, respectively, to correlate with high angiogenesis. Such a grading could be useful in predicting the degree of angiogenesis from biopsy material that is impossible to analyse reliably for microvessel counting. In that way, inoperable patients who are candidates for anti-angiogenic therapy could be recruited. The observation that high TPR does not define high angiogenesis in about half of the examined cases shows that TP may co-operate with other angiogenic factors or oncogene products for neovascularization to occur. Indeed, preliminary results (Koukourakis et al, 1996) showed a possible involvement of bcl-2 and c-erbB-2 oncoproteins in the TP-mediated angiogenic activity. A VEGF and TP synergy in defining angiogenesis in breast cancer has also been reported (Toi et al, 1995).

Survival analysis revealed that, although 'total thymidine phosphorylase reactivity' correlated with prognosis, cancer cell reactivity was a stronger indicator. This was because fibroblast TPR conferred an unusually good prognosis in low vascular grade cases (94% 5-year survival), and therefore cancer cell reactivity alone should be used as a more reliable prognostic indicator. In a multivariate model, excluding vascular grade and N stage, cancer cell TPR CCR proved to be an independent prognostic parameter. This may be used to assess prognosis in patients who are inoperable for medical reasons, in whom only bronchoscopic material is available.

We also examined the role of macrophages. We found high fibroblast TP expression reactivity with a high degree of macrophage infiltration regardless of the amount of stroma. Survival analysis showed that the degree of macrophage infiltration (per se) did not define different prognostic groups within the low and high vascular grade groups. Fibroblast TP overexpression determined a 5-year survival of up to 95% in the low vascular grade group. Loss (or absence) of fibroblast TP activity together with a high degree of macrophage infiltration in the low vascular grade group was associated with a very poor prognosis (30% 5-year survival). It may be that macrophages and fibroblasts have an important role in the early steps of tumour pathogenesis by inhibiting both angiogenesis and other mechanisms involved in the metastatic process. This hypothesis is supported by a recent study in breast cancer (Pupa et al, 1996), in which macrophage infiltration, although related to better prognosis, was also correlated with C-erbB-2 positivity. C-erbB-2 protein is well known to disrupt cell adhesion and to confer migratory abilities on cancer cells (Kanai et al, 1995). Of interest, in a recent study of ours, c-erbB-2 oncoprotein correlated with low vascular grade (Giatromanolaki et al, 1996*b*; Koukourakis et al, 1996).

As TP-reactive stromal fibroblasts and high macrophage infiltration was a common feature in low vascular grade cases, an anti-angiogenic role of combined macrophage and fibroblast activity in non-small-cell lung cancer is suggested. In a recent study in breast cancer, Leek et al (1996) showed that highly angiogenic areas were

© Cancer Research Campaign 1998

poorly populated with macrophages and macrophage-dense areas were poorly vascularized, which is in accordance with our observation. It was recently shown (Di-Pietro et al, 1993) that activated highly angiogenic macrophages produce the angiogenic inhibitor thrombospondin-1, which shows a complex angiogenic role of macrophages defined by positive and negative regulators. In a recent study (Dong et al, 1997), angiostatin expression by Lewis lung subcutaneously growing tumours required the presence of macrophages and was directly correlated with macrophage metalloelastinolytic activity. Fibroblasts have also a role in angiogenesis regulation. Dameron et al (1994) showed that fibroblasts also control angiogenesis through p53-mediated thrombospondin-1 gene regulation. It may be that activated fibroblasts represent a response to cytokines produced by tumour-infiltrating macrophages.

Our observations may also have therapeutic implications, suggesting that immunological manipulations that would restore the fibroblast and macrophage equilibrium within the tumour would have at least cytostatic activity. Interferon alpha (IFN-α) is a well-known cytokine that induces the expression of thymidine phosphorylase in cells (Schwartz et al, 1994). In a recent study, we found an induction of thymidine phosphorylase in vivo in lymphocytes of patients treated with escalating doses of IFN-α (unpublished data). On the other hand, granulocyte–macrophage colony-stimulating factor (GM-CSF) is a widely used growth factor that effectively stimulates the recovery of neutrophils and macrophages in patients undergoing chemotherapy and has been shown to be a potent activation signal for macrophages (Morissey et al, 1989). It would be of interest to examine whether the combination of IFN-α and GM-CSF restores the fibroblast and macrophage intratumoral activity and prevents tumour progression in refractory metastatic non-small-cell lung cancer. The recent observation that GM-CSF-stimulated macrophages have enhanced metalloelastinolytic activity, which is required for tumour angiostatin production, further supports such a therapeutic approach (Dong et al, 1997).

Briefly, we have provided evidence that fibroblasts and macrophages may have an important role in the pathogenesis of non-small-cell lung cancer. Their role is probably confined to early pathogenetic steps before the appearance of the angiogenic and migratory phenotype. The fact that not all angiogenic tumours are lethal and that not all non-angiogenic tumours have a good prognosis should not be attributed to random events, as factors such as fibroblast or macrophage activity may have a definitive role. Indeed, in our study, they defined groups with a 5-year survival as high as 95%. Further investigation is required to clarify the role of inflammatory cells in the angiogenic, migratory process and prognosis in non-small-cell lung cancer.

### ACKNOWLEDGEMENTS

We thank Dr J Vlachonicolis and E Mavromanolakis (Department of Biostatistics, School of Medicine, University of Crete) for their contribution in the statistical analysis of the study. We also thank Scheving Plough S.A. for financial support to cover the colour figure printing.

### REFERENCES

Craft PS, Harris AL (1994) Clinical prognostic significance of tumour angiogenesis. *Annals Oncol* **5**: 305–311

Dameron KM, Volpert OV, Tainsky MA and Bouck N (1994) Control of angiogenesis in fibroblasts by p53 regulation of thrombospondin-1. *Science* **265**: 1582–1584

Di-Pietro LA, and Polverini PJ (1993) Angiogenic macrophages produce the angiogenic inhibitor thrombospondin 1. *Am J Pathol* **143**: 678–584

Dong Z, Kumar R, Yang X and Fidler IJ (1997) Macrophage-derived metalloelastase is responsible for the generation of angiostatin in Lewis lung carcinoma. *Cell* **88**: 801–810

Eda H, Fujimoto K, Watanabe S, Ura M, Hino A, Tanaka Y, Wada K and Ishitsuka (1993) Cytokines induce thymidine phosphotylase expression in tumour cells and make them more susceptible to 5′-deoxy-5-fluorouridine. *Cancer Chemother Pharmacol* **32**: 333–338

Ferrara N, Houck K, Jakeman L and Leung DW (1992) Molecular and biological properties of vascular endothelial growth factor family of proteins. *Endocrine Rev* **13**: 18–32

Fontanini G, Bigini D, Vignati S, Basolo F, Mussi A, Lucchi M, Chine S, Angeletti CA, Harris AL and Bevilacqua G (1995) Microvessel count predicts metastatic disease and survival in non-small cell lung cancer. *J Pathol* **177**: 57–63

Fox SB, Moghaddam A, Westwood M, Turley H, Bicknell R, Gatter KC and Harris AL (1995) Platelet derived endothelial cell growth factor/thymidine phosphorylase expression in normal tissues – an immunohistochemical study. *J Pathol* **176**: 183–190

Fox SB, Westwood M, Moghaddam A, Compley M, Helen T, Whitehouse RM, Bicknell R, Gatter KC and Harris AL (1996) The angiogenic factor platelet-derived endothelial cell growth factor/thymidine phosphorylase is upregulated in breast cancer epithelium and endothelium. *Br J Cancer* **73**: 275–280

Giatromanolaki A, Koukourakis M, O'Byrne K, Fox S, Whitehouse R, Talbot D, Harris AL and Gatter KC (1996*a*) Prognostic value of angiogenesis in operable non-small cell lung cancer. *J Pathol* **179**: 80–88

Giatromanolaki A, Koukourakis M, O'Byrne K, Kaklamanis L, Dicoglou C, Trichia E, Whitehouse R, Harris AL and Gatter KC (1996*b*). Non small cell lung cancer: C-erb B-2 correlates with low angiogenesis and poor prognosis. *Anticancer Research* **16**: 3819–3825

Giatromanolaki A, Koukourakis M, Comley M, Kaklamanis L, Turley H, O'Byrne K, Harris AL and Gatter KC (1997) Platelet-derived endothelial cell growth factor (thymidine phosphorylase) expression in lung cancer. *J Pathol* **181**: 196–199

Guidi A, Abu-Jawdeh G, Berse B, Jackman RW, Tognazzi K, Dvorak HF and Brown LF (1995). Vascular permeability factor (vascular endothelial growth factor) expression and angiogenesis in cervical neoplasia. *J Natl Cancer Inst* **87**: 1237–1245

Horak ER, Russel L, Klenk N, LeJeune S, Smith K, Stuart N, Greenal M, Stepnewska K and Harris AL (1992) Angiogenesis, assessed by platelet/endothelial cell adhesion molecule antibodies, as indicator of node metastases and survival in breast cancer. *Lancet* **340**: 1120–1124

Ishikawa F, Miyazono K, Hellman U, Drexler H, Wernstedt C, Hagiwara K, Usuki, Takaku F, Risau W and Heldin CH (1989) Identification of angiogenic activity and the cloning and expression of platelet-derived endothelial cell growth factor. *Nature* **338**: 557–562

Kanai Y, Ochiai A, Shibata T, Oyama T, Ushijima S, Akimoto S and Hirohashi (1995) c-erbB-2 gene product directly associates with beta-catenin and plakoglobin. *Biochem Biophys Res Commun* **208**: 1067–1072

Klagsbrun M (1989) The fibroblast growth factor family: structural and biological properties. *Prog Growth Res* **1**: 207–235

Koukourakis M, Giatromanolaki A, Gatter KC and Harris AL (1996) Bcl-2 and c-erbB-2 gene products are involved in the regulation of thymidine phosphorylase (PD-ECGF) angiogenic activity in NSCLC. In *Proceedings of the 2nd International Congress on Lung Cancer,* No. 97A. 9–13 November 1996, Iraklion, Crete

Koukourakis MI, Giatromanolaki A, O'Byrne K, Comley M, Whitehouse R, Talbot DC, Gatter KC and Harris AL (1997) platelet-derived endothelial cell growth factor expression correlates with tumour angiogenesis and prognosis in non-small cell lung cancer. *Br J Cancer* **4**: 477–481

Leek RD, Lewis CE, Whitehouse R, Greenall M, Clarke J and Harris AL (1996) Association of macrophage infiltration with angiogenesis and prognosis in invasive breast carcinoma. *Cancer Res* **56**: 4625–4629

Moghaddam A and Bicknell R (1992) Expression of platelet-derived endothelial cell growth factor in *Escherichia coli* and confirmation of its thymidine phosphorylase activity. *Biochemistry* **31**: 12141–12146

Moghaddam A, Zhang HT, Fan TPD, Hu DE, Lees VC, Turley H, Fox SB, Gatter KC, Harris AL and Bicknell R (1995). Thymidine phosphorylase is angiogenic and promotes tumor growth. *Proc Natl Acad Sci USA* **92**: 998–1002

Mountain CF (1986). A new international staging system for lung cancer. *Chest* **89** (suppl): 225–233

 © Cancer Research Campaign 1998

Morrisey PJ, Grabstein KH and Reed SG (1989) Granulocyte/macrophage colony stimulating factor: a potent activation signal for mature macrophages and monocytes. *Int Arch Allergy Appl Immunol* **88**: 40–45

Parums DV, Cordell JL, Micklem K, Heryet AR, Gatter KC and Mason DY (1990) JC70: a new monoclonal antibody that detects vascular endothelium associated antigen on routinely processed tissue sections. *J Clin Pathol* **43**: 752–757

Patterson A, Zhang H, Moghaddam A, Bicknell R, Talbot D, Stratford I and Harris AL (1995) Increased sensitivity to the pro-drug 5′-deoxy-5-fluorouridine and modulation of 5′-fluoro-2′-deoxyuridine sensitivity in MCF-7 cell transfected with thymidine phosphorylase. *Br J Cancer* **72**: 669–675

Pupa SM, Bufalino R, Invernizzi AM, Andreola S, Rilke F, Lombardi L and Colnagli Menard S (1996) Macrophage infiltrate and prognosis in c-erbB-2 overexpressing breast carcinomas. *J Clin Oncol* **14**: 85–94

Schwartz EL, Baptiste N, O'Connor CJ, Wadler S and Otter BA (1994) Potentiation of the antitumor activity of 5-fluorouracil in colon carcinoma cells by the combination of interferon and deoxyribonucleosides results from complementary effects on thymidine phosphorylase. *Cancer Res* **54**: 1472–1478

Scott PAE and Harris AL (1994). Current approaches to targeting cancer using antiangiogenesis therapies. *Cancer Treat Rev* **20**: 393–412

Takanami I, Tanaka F, Hashizume T, Kikuchi K, Yamamoto Y, Yamamoto T and Kodaira S (1996). The basic fibroblast growth factor and its receptor in pulmonary adenocarcinomas: an investigation of their expression as prognostic markers. *Eur J Cancer* **32**: 1504–1509

Toi M, Hoshina S, Taniguchi T, Yamamoto Y, Ishitsuka H and Tominaga T (1995) Vascular endothelial growth factor and platelet derived endothelial cell growth factor are frequently coexpressed in highly vascularized human breast cancer. *Clin Cancer Res* **1**: 961–964

Usuki K, Saras J, Waltenberger J, Miyazono K, Pierce G, Thomason A and Heldin CH (1992). Platelet-derived endothelial cell growth factor has thymidine phosphorylase activity. *Biochem Biophys Res Commun* **184**: 1311–1316

Weidner N, Semple JP, Welch WR and Folkman J (1991). Tumor angiogenesis and metastasis – correlation in invasive breast carcinoma. *N Engl J Med* **324**: 1–8

© Cancer Research Campaign 1998